# Implementation of the Digital QS-SVM-Based Beamformer on an FPGA Platform

**DOI:** 10.3390/s23031742

**Published:** 2023-02-03

**Authors:** Somayeh Komeylian, Christopher Paolini

**Affiliations:** Electrical and Computer Engineering, San Diego State University, San Diego, CA 92182, USA

**Keywords:** digital beamforming, support vector machine, minimum variance distortionless response, linearly constrained minimum variance, direction of arrival estimation, FPGA, spatial filter, massive wireless communications

## Abstract

To address practical challenges in establishing and maintaining robust wireless connectivity such as multi-path effects, low latency, size reduction, and high data rate, we have deployed the digital beamformer, as a spatial filter, by using the hybrid antenna array at an operating frequency of 10 GHz. The proposed digital beamformer utilizes a combination of the two well-established beamforming techniques of minimum variance distortionless response (MVDR) and linearly constrained minimum variance (LCMV). In this case, the MVDR beamforming method updates weight vectors on the FPGA board, while the LCMV beamforming technique performs nullsteering in directions of interference signals in the real environment. The most well-established machine learning technique of support vector machine (SVM) for the Direction of Arrival (DoA) estimation is limited to problems with linearly-separable datasets. To overcome the aforementioned constraint, the quadratic surface support vector machine (QS-SVM) classifier with a small regularizer has been used in the proposed beamformer for the DoA estimation in addition to the two beamforming techniques of LCMV and MVDR. In this work, we have assumed that five hybrid array antennas and three sources are available, at which one of the sources transmits the signal of interest. The QS-SVM-based beamformer has been deployed on the FPGA board for spatially filtering two signals from undesired directions and passing only one of the signals from the desired direction. The simulation results have verified the strong performance of the QS-SVM-based beamformer in suppressing interference signals, which are accompanied by placing deep nulls with powers less than −10 dB in directions of interference signals, and transferring the desired signal. Furthermore, we have verified that the performance of the QS-SVM-based beamformer yields other advantages including average latency time in the order of milliseconds, performance efficiency of more than 90%, and throughput of nearly 100%.

## 1. Introduction

Adaptive digital beamformers have recently received substantial attention in a variety of applications such as MIMO wireless communications [1], fault detection and calibration [2], wireless localization [3], and mmWave communication systems [4]. The involved problems of the framework of the antenna array signal processing have mainly included techniques of beamforming and the DoA estimation. Techniques of the DoA estimation are employed for calculating the DoAs of sources, while beamforming algorithms are employed for tracking the desired signals in the real environment and nulling out other interference signals. In other words, techniques of the DoA estimation provide a nonlinear mapping from original signal sources to measured results in outputs of array elements, while beamforming techniques have focused on recovering the original signals from interested sources.

Beamforming techniques can perform using any type of optimization method, or one of two well-established techniques of MVDR and LCMV, or a combination of the aforementioned techniques. Although the MVDR beamforming technique is capable of determining optimal weight vectors for its beamsteering performance, its nullsteering performance remains unsatisfactory. However, the LCMV beamforming technique can reduce interference signals without losing the information strength. Compared to the MVDR beamforming technique, the LCMV beamforming method offers several different advantages of (1) better performance in suppressing interference signals, (2) a significant reduction in side lobe level, and (3) a drastic increase in signal-to-interference noise ratio (SINR). Hence, in this work, the beamforming functionality has been fulfilled using a combination of the LCMV and MVDR techniques to take advantage of both beamforming techniques and thereby achieve more robustness and stability in the performance of the proposed digital beamformer. The LCMV beamforming technique in the real environment has been performed for suppressing interference signals or improving the nullsteering functionality while the MVDR beamforming technique updates weight vectors for the beamsteering on the FPGA board.

Therefore, in this case, the LCMV beamformer is capable of providing a higher directivity and lower sidelobe level in addition to determining optimal weight vectors.

The DoA estimation has been fulfilled based on the QS-SVM method with a small regularizer. The practical implications of kernel-based classifier techniques, such as the SVM methods, are mostly limited only to supporting linearly-separable datasets. However, almost all practical problems have inherent non-linear characteristics. To address this limitation, feasible methods for practical applications have to be capable of supporting non-linearly separable datasets without performing a mapping to a larger feature space. In other words, we are interested in developing feasible techniques, which include non-linear classifiers in the original spaces of their own datasets. The QS-SVM model [5] employs quadratic surfaces for classifying a non-linear dataset in its original space without mapping it to a higher dimensional feature space. Although the highly non-linear classification generated by the Gaussian kernel function or quadratic surfaces performs more effectively than the SVM techniques based on hyperplanes in terms of classification accuracy, we can still improve the performance of SVM techniques by producing hyperplane classifiers in the conventional QS-SVM models. The QS-SVM technique does not produce any classifier hyperplane for capturing linearly-separable datasets. To overcome this limitation, we have added a regularizer into the objective function of the QS-SVM model results, as discussed in detail in [5,6]. The presence of hyperplanes yields an increase in the number of outliers in the training datasets, and thereby the QS-SVM model outperforms other models in terms of the generalization capability and robustness to outliers in addition to capturing linearly-separable datasets. A relatively large value of the penalty parameter of the regularizer can capture linearly-separable datasets. Furthermore, an appropriate choice of a small value of the penalty parameter of the regularizer results in more quadratic surfaces.

Maximizing SINR values is dependent on not only an increase in the gain in the directions of desired signals but also an enhancement in the spatial distribution of a radiation pattern of the antenna array. Hence, to further improve the beamforming performance, the antenna array has to be capable of having the horizontal and vertical spatial distribution of nulls of the radiation pattern to obtain an additional degree of freedom for suppressing interference. Indeed, beamforming techniques have been used as a spatial filter for reception and transmission by the antenna array.

As described in details in [7], the hybrid antenna has demonstrated superior advantages over other available antenna arrays for massive wireless communications [7]. The proposed techniques for beamforming and the DoA estimation are strongly capable of performing in real-time on hardware implementations or on an FPGA board.

FPGA technology has recently received tremendous attention as it offers several advantages over other available embedded processors, such as digital signal processing (DSP) and specific application integrated circuit (ASIC) devices [8,9]:

- Recent FPGA technology has programmable logic and the capability of algorithm parallelization for further enhancing power consumption, flexibility, and accuracy.
- Recent advances in the FPGA architectures include a higher storage density, a drastic reduction in power consumption and cost, a large number of gates, and a high-performance processor.
- Recent FPGA software and high-level optimizations have to be accompanied by architectural changes in the FPGA board in order to satisfy drastic computations of SVM-based applications. Advances in FPGA technology have rigorously presented high-level software tools to be easily adjusted to the FPGA hardware.

Furthermore, FPGAs have evolved over the past several years into more heterogeneous devices integrated with various types of “hard IP” blocks, such as PCIe solid-state drives (SSDs), floating-point DSPs, etc. Hard blocks can also be implemented in FPGAs to address specific applications.

In this work, we have presented the implementation setup of the digital beamforming on the FPGA platform when performing the QS-SVM modeling for the DoA estimation using the proposed hybrid antenna array with bowtie elements. The deployment set-up of the proposed digital beamformer on the FPGA board consists of the software and hardware implementations. The software implementation involves the LCMV beamformer technique for performing nullsteering of the hybrid antenna array in a real environment, while the hardware deployment involves implementing a quadrature programming solver for the QS-SVM technique with a regularizer, weight updating using the MVDR technique, and inner multiplications of the QS-SVM on the FPGA platform.

In summary, the novel research contributions of this work are as follows:

- For the first time, the QS-SVM-based beamformer has been implemented using the hybrid antenna array with bowtie elements on an FPGA board.
- For the first time, this work presents an implementation of the proposed digital beamformer in both the real environment and hardware board.
- The implementation of the QS-SVM optimization method for the DoA estimation on an FPGA board has been rigorously demonstrated for the first time.
- We have achieved a superior performance of the digital QS-SVM-based beamformer in terms of beamforming, nullsteering, and beamsteering.
- A performance evaluation of the QS-SVM-based beamformer has been fulfilled in terms of throughput, latency, and performance efficiency.

Consequently, in addition to the introduction section, this work is organized into the seven following sections:

Section 2 is allocated for reviewing the related technical literature. The methodology and theoretical framework of the QS-SVM-based digital beamformer has been provided in Section 3. We have described the theoretical and mathematical formulations of the proposed method for the DoA estimation in the proposed digital beamformer in Section 4. The deployment setup of the digital QS-SVM beamformer in the real environment (or here in the software) and hardware environment (or here on the FPGA board), has been presented in Section 5. We have assumed that three sources in the directions of $30^{\circ }$, $50^{\circ }$, and $45^{\circ }$ and 5 hybrid array antennas are available. The QS-SVM-based digital beamformer has been deployed for the DoA estimation. In Section 6, we have demonstrated the research project deliverables of the digital QS-SVM beamformer. The excellent effectiveness, performance, and reliability of the proposed digital beamformer in terms of spatial filtering, even under noisy conditions, has been verified in this section. In Section 7, we have evaluated and validated the digital beamforming performance. In this sense, results-based monitoring allows for providing a framework to assess and evaluate the proposed digital beamforming performance in terms of (1) throughput evaluation, (2) average latency time, and (3) performance efficiency. The conclusions of this work have been presented in Section 8.

## 2. Literature Review and Related Work

The performance of deploying a digital beamforming technique is significantly affected by the hardware platform, hardware design, and system architecture of a beamformer, and the involved optimization methods. Hence, the discussion of this section presents a brief review of the technical literature on implementing digital beamforming on FPGA accelerated hardware platforms.

Dick et al. in [10] have proposed the real-time QR decomposition (QRD)-based beamforming method on an FPGA platform. However, the significant disadvantage of the proposed beamforming technique consists in determining optimal weights using the MVDR technique, without performing any nullsteering method. The MVDR technique is capable of performing beamsteering by determining the weight vectors; however, its nullsteering performance remains unsatisfactory. In this sense, when a mismatch occurs between the direction of the steered main lobe and the direction of the signal of interest, the MVDR beamformer considers the reference signal as an interference signal and thereby strongly dissipates the signal of interest. Hence, in a wireless communication channel with an inherent characteristic of strong multi-path effects, we have to perform a beamforming technique with the nullsteering capability in addition to determining optimal weights such as the LCMV beamformer technique. Furthermore, in [10], the QRD algorithm has been employed for computing weights on an FPGA platform. However, it may not overcome the challenges of a massive wireless communication channel since beamforming techniques in every wireless communication channel involves computationally-intensive operations on large volumes of data. Therefore, we have to implement different strong optimization methods and beamformer techniques on a hardware platform to achieve satisfactory performance.

The implementation of an adaptive digital beamforming technique using the conventional least mean squared (LMS) method on the FPGA hardware for massive antenna arrays has been proposed in [11]. A major disadvantage of the proposed adaptive digital beamforming method consists of the dependency of the convergence speed of the conventional LMS on a spread of eigenvalues of the correlation matrix of the input signal. In other words, when the input signal consists of disparate eigenvalues, slow modes of convergence are dominant. This negative effect worsens the adaptive digital beamforming performance by increasing the number of antennas [11]. Although the adaptive digital beamforming performance in terms of throughput, latency, and energy consumption has significantly enhanced, the strong mutual coupling effect between 64 antennas yields a performance degradation in terms of antenna efficiency, which is not reported in [11]. In addition to the aforementioned disadvantages, the functionality of spatial filtering of the digital beamforming technique and its performance evaluation have not been demonstrated in [11].

The Cholesky decomposition or factorization has been performed on the input covariance matrix and results have shown a good performance under the computational complexity in [12]. However, the obtained results and efficiency of Cholesky decomposition differ considerably in terms of the implementation and architectural design parameters of the computing hardware, as reported in table 2 of [12]. Furthermore, the rectangular antenna array in [12] is very sensitive to the mutual coupling effect compared to a circular antenna array.

Xin et al. in [13] implemented a digital beamforming technique on an FPGA with performing a DSP processor. They have employed the Wiener vector solution for estimating the minimum mean squared error by minimizing a time-averaged squared error function. Although the Wiener vector solution results in a drastic noise reduction, the involved solutions include computing partial derivatives with respect to both the real and imaginary parts and require both parts to be zero. Hence, its hardware implementation involves computational complexity, high operational costs, and low computational speed, which is not of practical interest in wireless communication channels. Ullah et al. in [14] proposed a millimeter-wave digital beamforming technique at the receiver on an FPGA platform to ameliorate interference signals between two adjacent channels in the MIMO wireless communication channel. Although they could achieve superior performance in terms of maximum SIR values of approximately 36 dB in channel #1, and 26 dB in channel #2, a major disadvantage of the proposed beamformer in [14] includes the sensitivity of the design parameters of its hardware platform to the practical imperfections and losses in coaxial cables, the custom-designed encoder circuit board, and the adapter board.

## 3. Proposed Methodology and Techniques for the Spatial Signal Processing

The framework of array signal processing refers to spatial signal processing, which is composed of designing an appropriate configuration of the antenna array for steering the main lobe of its radiation pattern in an arbitrary direction of space, and then implementing beamforming techniques for placing nulls of its radiation pattern towards interference signals while steering (or producing) the main lobe of its radiation pattern towards targets (or users) based on the measured weight vectors. Therefore, in the following work, we have demonstrated the proposed configuration of the antenna array, and then, described the proposed beamforming techniques.

### 3.1. Hybrid Antenna Array

The configuration of any antenna array drastically affects the characteristics of the main lobe of its radiation pattern. Hence, the preliminary stage of any beamforming technique includes choosing an appropriate antenna array. Each of antenna arrays has its own constraints and advantage, as discussed in detail in [7]. A uniform rectangular array (URA) antenna has a weak performance with a low resolution. Furthermore, the radiation pattern of any URA consists of having an additional major lobe on the opposite side of its main lobe with the same intensity.

Although the circular antenna array does not have any edge elements, its radiation pattern includes grating lobes and sidelobes. Moreover, since the radiation pattern of the circular antenna array does not include any null in the azimuth planes, it has a weak nullsteering performance.

In this work, we have proposed the hybrid antenna array with bowtie elements, whose radiation pattern includes horizontal and vertical nulls and a narrower main lobe compared to the other available antenna arrays [7]. Hence, we can efficiently steer its maximum antenna gain (or main lobe) toward targets and place its nulls toward interference signals by utilizing beamforming techniques. In other words, since the radiation pattern of the 3D hybrid antenna array consists of deep nulls, it can outperform in terms of suppressing interference signals in addition to its strong steering performance in tracking targets (or users) by implementing beamforming techniques. Therefore, the hybrid antenna array with bowtie elements outperforms its geometrical counterpart with dipole elements [7], and other available antenna arrays due to the distinct features of its bowtie elements, and geometrical configuration.

### 3.2. Methodology and Theoretical Framework

**Figure 1:**
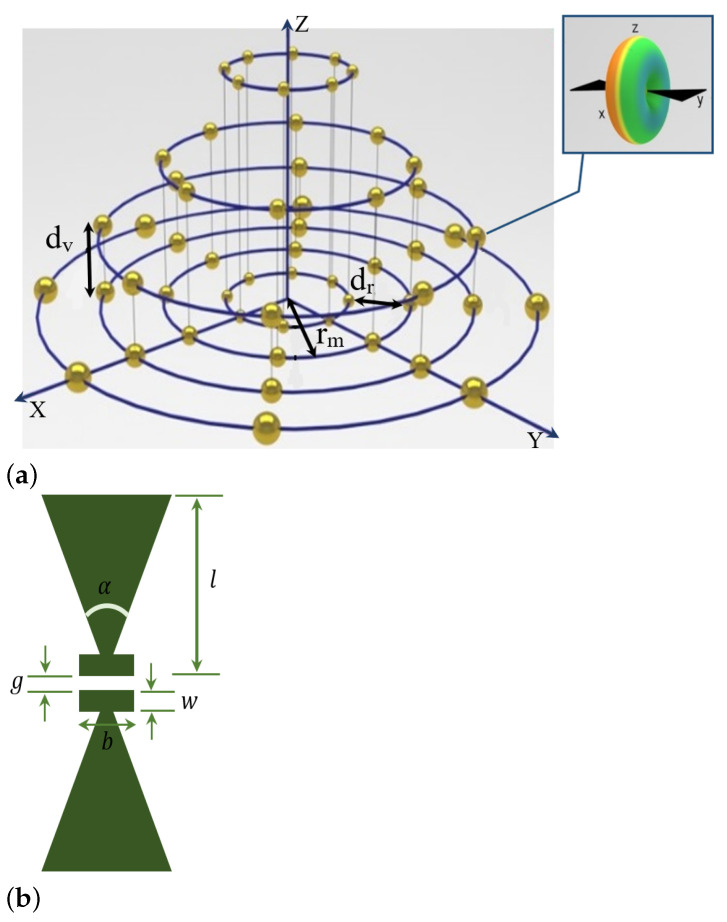
A demonstration of the 3D configuration of the hybrid antenna array with bowtie elements at the frequency of the operation of 10 GHz with bowtie elements. (**a**) The assigned design parameters of the hybrid antenna array are listed in detail in Table 1. In this work, each hybrid antenna array consists of three cylindrical antenna arrays [15], and one circular antenna array [16], and (**b**) a top view of the bowtie antenna; *l*: arm length, α: flare angle, *g*: feed gap, *w*: bar width, *b*: bar length. In this work, the design parameters of each bowtie element are assumed to be *l* = 6 mm, $\alpha =60^{\circ }$, *g* = 0.02 mm, *w* = 0.02 mm, and its thickness is equal to 0.01 mm [7].

**Figure 2:**
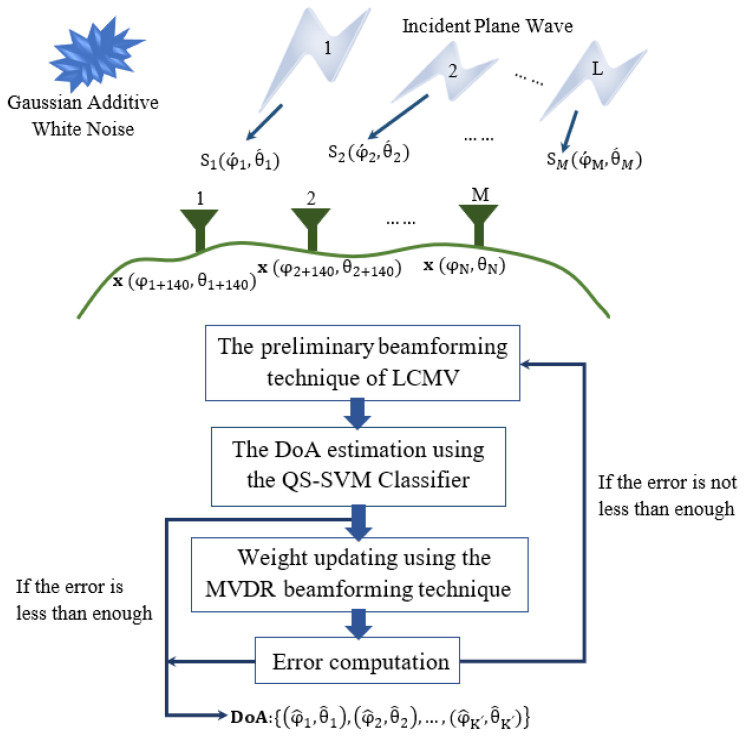
Procedures for the DoA estimation using the QS-SVM-based digital beamformer. It is assumed that there are *M* numbers of the proposed hybrid array antennas in Figure 1b of [7]. Each hybrid antenna array consists of 140 elements whose outputs have been measured. The proposed QS-SVM algorithm for the DoA estimation measures outputs of *N* array elements and predicts the directions of *L* signals impinging on the array elements. In this figure, position vectors are supposed to be in a two-dimensional plane of θ and ϕ. The process of the DoA estimation is to monitor the *N* outputs of antenna elements and predict the angle of arrival of *L* signals, l=1,2,…,L. N=M×140. M = 5 in this work. $K^{\prime }$ denotes a number of sources that we can obtain after the DoA estimator.

In the previous section, we proposed the hybrid antenna array with bowtie elements due to its superior performance in terms of a very directive radiation pattern, the SINR values, and the antenna efficiency, as demonstrated in [7]. Discussion in this section has focused on presenting an outline of the theoretical and mathematical framework of the QS-SVM-based digital beamformer using the hybrid antenna array with bowtie elements of Figure 1, as demonstrated in Figure 2. The collected data in outputs of bowtie elements of the 5 hybrid array antennas results from different available sources including unwanted and desired signals in the real environment. To overcome the dominant challenge of the presence of unwanted signals including interference, noise, etc. in the real environment, we have preliminary implemented the LCVM beamforming technique due to its strong nullsteering performance in suppressing the unwanted signals.

The digital beamformer is also capable of performing the QS-SVM optimization method for the DoA estimation of the measured outputs of bowtie elements of the hybrid array antennas, Figure 2. The QS-SVM technique is capable of overcoming high computational complexities and transferring very large volumes of data in massive wireless communications. We have demonstrated that the proposed methodology and techniques are well-matched for beamforming applications due to their simplicity to be implemented on the hardware platform as well as their superior performance to overcome the limitations of modern wireless communication channels, especially the multi-path effect.

## 4. Methods of Modeling and Producing Data

In the real environment, we assumed that the available sources emit sinusoidal signals. Data modeling is the process of producing data based on assumptions of the real environment to generate signal sources. In this study, we have employed the MATLAB programming platform to provide raw data. Since Mathworks functions of the MATLAB programming platform have been extensively tested, evaluated, and verified based on IEEE standards and criteria, our obtained simulation results through MATLAB provide a very high level of realistic accuracy. In the preliminary stage of producing data, a sinusoidal function is considered as an available signal. In this scenario, the available signal is significantly affected by environmental noise, the coupling effect, and communication channel conditions. In this study, conditions of the communication channel are supposed to be the Ricean fading channel, line of sight (LoS), and equal correlation matrices, as described in detail in [17].

In addition, the additive white Gaussian noise (AWGN) is added to the simulated signal by the standard Mathworks’ function of AWGN. Afterward, the standard Mathworks function of *collectPlaneWave()* exerts the plane wave condition to the simulated signal or incoming signal to the hybrid antenna array, which has experienced the AWGN noise and channel conditions. In this work, the white Gaussian noise has a variance of $\sigma^{2}$. The noise model of v consists of the covariance matrix of $\sigma^{2}I$, which is a centered complex vector.

### 4.1. The Proposed Beamforming Technique

Signal measurements at the outputs of array elements of the hybrid antenna array are modeled by random vectors. Ultimately, a beamforming model consists of parameters, such as the source power and covariance of the noise power. As demonstrated in Figure 2, the incident plane wave is expressed in terms of its generated source and locations in which measured.

The beamforming technique is employed for calculating the scalar product between the measured data at outputs of array elements and the steering vector in the following equation,

(1) $${\langle R_{n}\left(\phi_{n},\theta_{n}\right),h_{n}\left(\phi_{n},\theta_{n}\right)\rangle |}^{2}$$

where n=1,2,…,N. Equation (2) represents the mean of the estimated power of the source,

(2) $$R_{n}\left(\phi ,\theta \right)=\sum_{n=1}^{N}\left(\underset{\mathrm{source}}{\underset{︸}{C_{n}e^{jK.r_{n}}}}+\underset{\mathrm{noise}}{\underset{︸}{v_{n}}}\right)$$

where $v_{n}=\sigma^{2}I$ for n=1,2,…,N. The steering vector of the proposed hybrid antenna array in [7] is obtained by the multiplication of the steering vector matrices of the three cylindrical antenna arrays and circular antenna array in the following equations [18],

(3) $$\begin{array}{c} h_{n}^{\left(T\right)}\left(\phi ,\theta \right)=\underset{\mathrm{for}\;\mathrm{the}\;\mathrm{three}\;\mathrm{cylindrical}\;\mathrm{antenna}\;\mathrm{arrays}}{\underset{︸}{\frac{1}{N^{\left(1\right)}}\frac{C_{n}^{\left(1\right)}\left(\phi ,\theta \right)}{|C_{n}^{\left(1\right)}\left(\phi ,\theta \right)|}⊗\frac{1}{N^{\left(2\right)}}\frac{C_{n}^{\left(2\right)}\left(\phi ,\theta \right)}{|C_{n}^{\left(2\right)}\left(\phi ,\theta \right)|}⊗\frac{1}{N^{\left(3\right)}}\frac{C_{n}^{\left(3\right)}\left(\phi ,\theta \right)}{|C_{n}^{\left(3\right)}\left(\phi ,\theta \right)|}}}\\ ⊗\underset{\mathrm{for}\;\mathrm{the}\;\mathrm{circular}\;\mathrm{antenna}\;\mathrm{array}\;(\mathrm{when}\;h=0)}{\underset{︸}{\frac{1}{N^{\left(4\right)}}\frac{C_{n}^{\left(4\right)}\left(\phi ,\theta \right)}{|C_{n}^{\left(4\right)}\left(\phi ,\theta \right)|}}} \end{array}$$

in which the two-dimensional steering vector of each sub-array of the hybrid antenna array in terms of the phase of the coefficients of the source vector is given by,

(4) $$h_{n}\left(\phi ,\theta \right)=\frac{1}{N}\frac{C_{n}\left(\phi ,\theta \right)}{|C_{n}\left(\phi ,\theta \right)|}$$

where coefficients of the source vector are expressed by

(5) $$C\left(\phi ,\theta \right)=\left[\begin{array}{c} g_{1}\left(\phi ,\theta \right)e^{-\frac{j2\pi }{\lambda }\left(r\sin\theta \cos\left(\theta -\theta_{1}\right)+h\cos\phi \right)}\\ g_{2}\left(\phi ,\theta \right)e^{-\frac{j2\pi }{\lambda }\left(r\sin\theta \cos\left(\theta -\theta_{2}\right)+h\cos\phi \right)}\\ \vdots \\ g_{N}\left(\phi ,\theta \right)e^{-\frac{j2\pi }{\lambda }\left(r\sin\theta \cos\left(\theta -\theta_{N}\right)+h\cos\phi \right)} \end{array}\right]$$

(6) $$K=\frac{2\pi }{\lambda }\left(K_{x},K_{y},K_{z}\right)=\frac{2\pi }{\lambda }\left(\sin\phi \sin\theta ,\sin\phi \cos\theta ,\cos\phi \right)$$

where K represents the wave vectors in directions of radius vectors of $r_{n}$ pointed to *n*th array elements. Here, position vectors are assumed to be in a two-dimensional plane of θ and ϕ,

(7) $$r_{n}^{T}={\left[r\cos\theta_{n},r\sin\theta_{n},h\right]}^{T}.$$

Hence, the phase shift relative to the origin is given by,

(8) $$\begin{array}{c} K.r_{n}=\frac{2\pi }{\lambda }\left[\sin\phi \sin\theta \;\sin\phi \cos\theta \;\cos\phi \right]\left[\begin{array}{c} r\cos\theta_{n}\\ r\sin\theta_{n}\\ h \end{array}\right]=\\ \;\;\;\;\;\;\;\;\;\;\;\;\frac{2\pi }{\lambda }\left(r\sin\phi \cos\theta \cos\theta_{n}+r\sin\phi \cos\theta \sin\theta_{n}+h\cos\phi \right) \end{array}$$

Furthermore, $g_{n}\left(\phi ,\theta \right)$ refers to the antenna gain at the *n*th element.

### 4.2. The QS-SVM Technique for the DoA Estimation

In this section, we have provided the theoretical and mathematical procedures for the DoA estimation using the kernel-free quadratic SVM method. The QS-SVM model is proposed for direct classification by the quadratic function, which is applicable to linear and non-linear datasets.

**The training procedure:** The supervised learning algorithms are designed to construct a model within the training phase. The machine learning algorithm analyzes the training dataset to generate parameters for providing quadratic classifier surfaces such that a maximum margin engenders between each of the two different classes of data. Therefore, the output of the training procedure is a model trained by the training dataset. Suppose a non-linearly separable training dataset with correct values of outputs are in pair with correct values of inputs in the following form,

(9) $$I={\left\{x_{i},y_{i}^{c}\right\}}_{i=1,c=1}^{i=n,q=Q}$$

Equation (9) holds for the *n* index of the training data points and the *Q* number of classes. And, also the training labels are defined in the following equation,

(10) y=i∈{1,2,⋯,G}

where *G* refers to a number of output labels. During the training procedure, the QS-SVM algorithm not only generates the hyperplane classifiers but also updates parameters and hyperparameters (w,b,c) for changing the locations of hyperplanes so that two classes with the largest geometrical margin can be separated. In other words, we can obtain the QS-SVM model by maximizing the sum of the relative geometrical margins at all training points with respect to f(x)=0,

(11) $$f\left(x\right):\frac{1}{2}{\left(x^{i}\right)}^{T}wx^{i}+b^{T}x^{i}+c$$

Equation (11) represents quadrature classifiers with the largest margin between each of two different closest classes, and w and *c* represent the weight vectors perpendicular to the quadrature classifiers, and the bias value for shifting the quadrature classifiers parallel to themselves, respectively [19]. In other words, the proposed quadratic surfaces of Equation (11) are employed for separating *n* training points of the given training dataset. The machine learning algorithm analyzes the training dataset and constructs quadratic classifier surfaces from the training dataset such that the sum of the relative geometrical margins becomes maximum at all training points such that no training point appears between each of the two different closest classifier surfaces, as described in Equations from (12) to (16).

By minimizing

(12) $$\min\sum_{i=1}^{n}{∥wx^{i}+b∥}_{2}^{2}+\hat{\eta }\sum_{i=1}^{n}\xi_{i}$$

subject to constraints:

(13) $$\begin{array}{c} y^{i}\left(\frac{1}{2}{\left(x^{i}\right)}^{T}wx^{i}+b^{T}x^{i}+c\right)\geq 1-\xi_{i}, \end{array}$$

(14) $$\begin{array}{c} \xi_{i}\geq 0,\;i=1,\ldots ,n, \end{array}$$

(15) $$\begin{array}{c} w=w^{T}\in R^{m\times m}, \end{array}$$

(16) $$\begin{array}{c} \left(b,c\right)\in R^{m}\times R^{1}. \end{array}$$

where R refers to the set of real numbers. $R^{m}$ represents the *m*-dimensional Euclidean space. $R^{m\times m}$ is the space of all m×m matrices.

In a condition, in which the training dataset cannot be quadratically separated or f(x)=0, training points should satisfy one of the following conditions:

**Condition #1:** $\;x^{i},y^{i}=-1,$

  but

(17) $$\frac{1}{2}{\left(x^{i}\right)}^{T}wx^{i}+b^{T}x^{i}+c>-1,$$

**Condition #2:** $\;x^{i},y^{i}=+1,$

  but

(18) $$\frac{1}{2}{\left(x^{i}\right)}^{T}wx^{i}+b^{T}x^{i}+c<+1.$$

**The testing procedure:** The testing dataset, or the remainder of the dataset which was not employed for the training dataset, is fed to the model. As soon as the testing dataset is fed to the model, the model becomes fixed such that it cannot change anymore. Then, the quadratic classifier surfaces are used to separate data points of the testing dataset. In other words, to assess how well the model can process the real-world data and generate accurate predictions, we employ the unseen dataset, or testing dataset. In the mathematical sense, the decision function of $D_{ij}\left(x\right)$ can be expressed by,

(19) $$D_{ij}\left(x\right)=\frac{1}{2}{\left(x\right)}^{T}w_{ij}x+b_{ij}x+c_{ij}$$

where $D_{ij}\left(x\right)=-D_{ji}\left(x\right)$. Therefore, the quadratic surface is applied to decide which class each data point of the testing dataset belongs to.

(20) $$D_{i}\left(f\left(x\right)\right)=\sum_{i=1,j\neq i}^{n}sign(D_{ij}\left(x)\right)$$

where

(21) $$sign\left(x\right)=\left\{\begin{array}{cc} 1 & \mathrm{for}\;x\geq 0\\ -1 & \mathrm{for}\;x<0 \end{array}\right.$$

## 5. Implementation Setup of the QS-SVM-Based Beamformer on the FPGA Board

**Figure 3:**
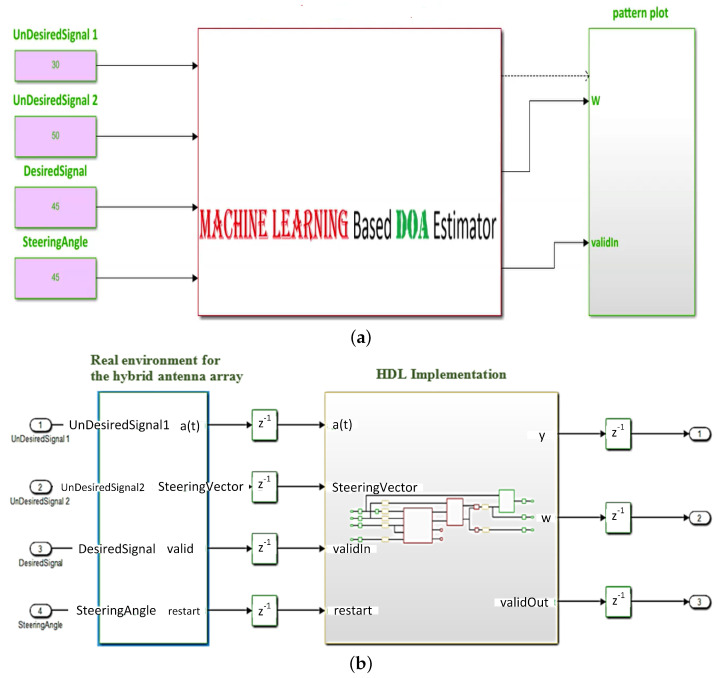
(**a**) A demonstration of the proposed QS-SVM-based digital beamformer. Here, position vectors are assumed to be in a plane of θ variable and ϕ constant. An angle of $45^{\circ }$ is the direction of a desired signal, and the steering angle should be identical to it. However, angles of $30^{\circ }$, and $50^{\circ }$ represent the directions of undesired signals, and (**b**) a demonstration of the proposed QS-SVM-based digital beamformer in the real and hardware environments.

The discussion in the previous sections has focused on providing the involved theoretical and mathematical techniques and solutions to implement the proposed QS-SVM beamformer. In this section, we have represented the efficient and compact hardware implementations of the proposed approaches and solutions for the DoA estimation and beamforming techniques used in the QS-SVM beamformer. The technical approaches and solutions to deploy the proposed digital beamformer on the FPGA platform have been employed for two different environments: the real environment as we have simulated it in the software environment and the hardware environment or here the FPGA board, as demonstrated in Figure 3.

### 5.1. Real Environment and Software Implementation

Incident signals impinging on elements of the hybrid antenna should be magnified and down-converted to any convenient intermediate range of frequency. Phase references, or here phases of desired signals, remain unchanged for all operations of front ends. Then, the signal output of any front end should be digitalized by the A/D converter.

The performance of the proposed QS-SVM-based beamformer would be further clarified by performing the FPGA-based implementation for a practical example in the real environment, Figure 3. It is assumed that the direction of the desired source is $45^{\circ }$, but three signals in directions of $45^{\circ }$, $30^{\circ }$, and $50^{\circ }$ impinge on the five available hybrid array antennas. The direction of the desired source is $45^{\circ }$. Only one of three signals, which impinged on the hybrid antenna arrays, is aligned with the direction of the desired source. In this sense, the LCMV adaptive beamforming technique could significantly cancel interference signals and the MVDR technique could strongly steer the main lobe of the radiation pattern of the antenna arrays toward the direction of the desired source. Equations (11) to (19) have demonstrated mathematical operations and spatial constraints involved with the proposed hybrid antenna array on the FPGA board. Angles of impinging signals have to be adaptively computed by the preliminary LCMV beamforming technique in the real environment. Beamforming techniques based on recognizing spatial locations of signals are substantially degraded by imperfections of array elements, mutual coupling, pointing errors, and multi-path effects in wireless communications. Especially, the LCMV algorithm may fail to perform appropriately when desired signals are expanded in all directions due to multi-path effects.

### 5.2. Hardware Environment and FPGA Implementation

**Figure 4:**
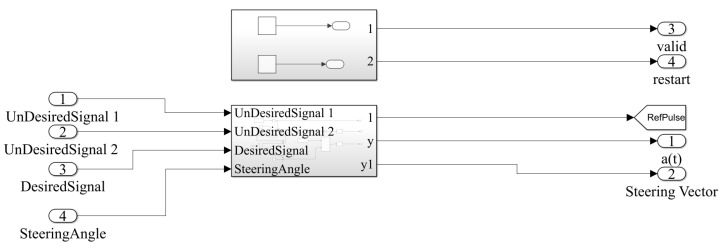
A demonstration of the spatial LCMV beamforming technique in the real environment integrated with the FPGA blocks. The direction of the desired source is $45^{\circ }$. However, two other directions of $50^{\circ }$ and $30^{\circ }$ impinge on the hybrid array antennas as unwanted signals. The LCMV beamforming technique is capable of spatially filtering unwanted signals.

**Figure 5:**
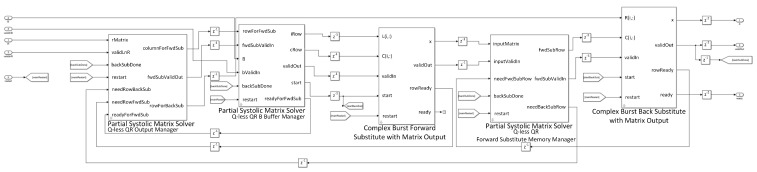
A demonstration of the complex Q-less QR forward-backward substitute on the FPGA board. In this work, Quadratic programming (QP) has been employed for solving the QS-SVM algorithm. The QP solver refers to the mathematical problem of finding weight vectors w in Equation (11) with respect to f(x)=0 and under the aforementioned constraints of Equation (17) and (18).

**Figure 6:**
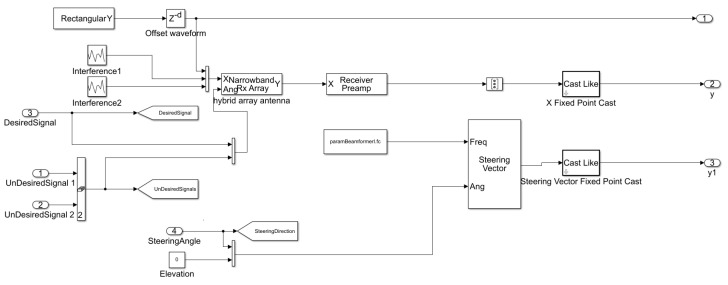
The aforementioned subsystem in Figure 4, the LCMV beamforming technique, and other mathematical operations in the real environment. The Simulink demonstration of all mathematical operations in the MATLAB platform with detail.

**Figure 7:**
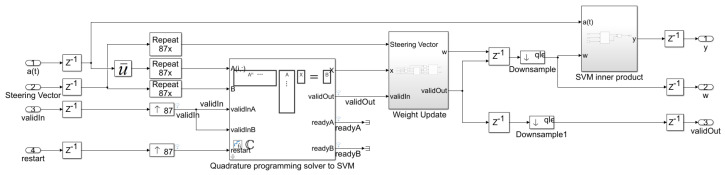
Hardware implementation of the proposed digital SVM-based beamformer on the FPGA platform.

**Figure 8:**
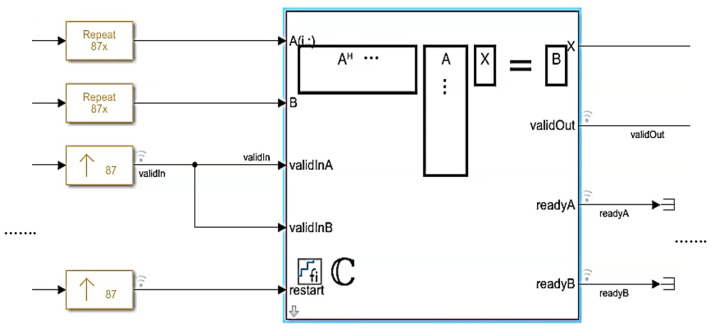
The quadrature programming solver for solving the QS-SVM problem in the HDL implementation.

**Figure 9:**
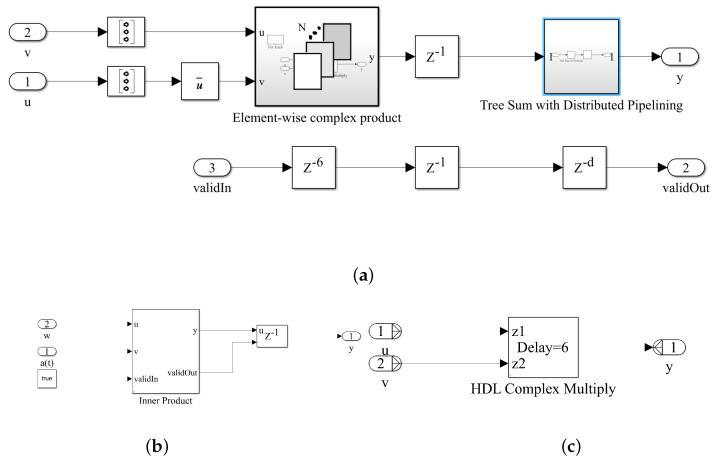
(**a**) A hardware demonstration for the inner products in the QS-SVM-based beamformer using HDL, (**b**) QS-SVM inner product, and (**c**) HDL complex multiplication.

**Figure 10:**
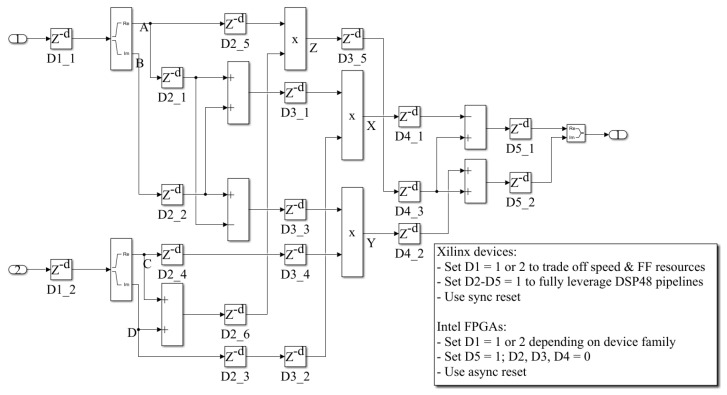
The hardware implementation of HDL complex multiplications of the QS-SVM optimization method.

**Figure 11:**
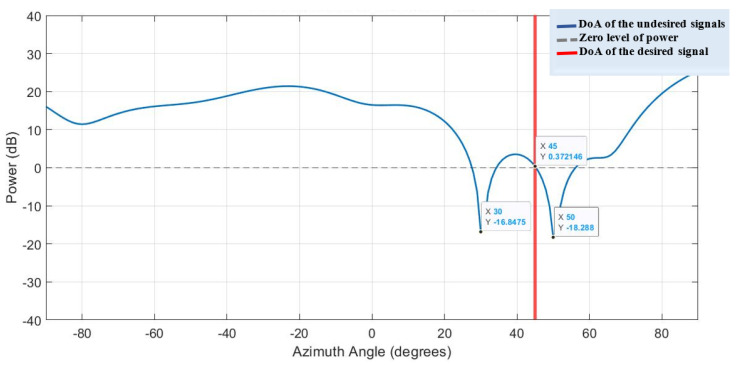
The spatial filtering performance of the QS-SVM-based digital beamformer on the FPGA board. The obtained results are associated with the part of the pattern plot in Figure 3.

The proposed beamformer technique for the real environment is very sensitive to multi-path effects, which is an inherent property of wireless communication channels. Therefore, in this section, a further enhancement in the performance of the QS-SVM-based beamformer has been achieved by the hardware implementation. All MATLAB codes of the proposed QS-SVM-based beamformer, including data collection codes, the LCMV, and MVDR beamforming codes, the proposed hybrid antenna array codes, and codes of the QS-SVM optimization method, have to be efficiently programmed into a hardware description language (HDL) in the format of arithmetic operations, such as multiplications and/or additions. Three main parts of the hardware implementation of the QS-SVM-based digital beamformer on the FPGA platform, including quadrature programming solver, weight updating, and SVM inner multiplications, have been rigorously demonstrated in Figure 3, Figure 4, Figure 5, Figure 6, Figure 7, Figure 8, Figure 9, Figure 10 and Figure 11.

**Quadrature Programming Solver:** The SVM algorithm allows for estimating a function, which maps the input data to a finite set of output labels [5,6] as discussed in detail in the previous section. Hence, the QS-SVM-based beamformer can overcome all constraints of the spatial reference techniques and achieve superior performance in terms of SINR. There is no information about the probability distribution of the input data. Therefore, we have to minimize errors between the array output and the reference signals (or here desired signals) for the number of available data in the left-hand side of Equation (22),

(22) $$R\left(f\right)=\int_{\Omega }L\left(f\left(x_{i}\right),y_{i}\right)\;dP\left(x_{i},y_{i}\right)\approx \sum_{i=1}^{N}w_{i}\;L\left(f\left(x_{i}\right),y_{i}\right)$$

where $\Omega =R^{m\times m}$ and *L* refers to the loss function.

We have employed the approach of Q-less QR decomposition with a forgetting factor to deploy the QS-SVM-based beamformer on the FPGA hardware. In the preliminary stage, the integral of a continuous function, based on quadrature programming, has been expended in terms of a superposition of the weighted loss function at available data points, as represented in the right-hand sight of Equation (22). As demonstrated in Figure 5, we have rigorously produced $f(x_{i})$ using the approach of Q-less QR decomposition, as described in detail [6,20]. When an upper triangular factor becomes ready, we have computed the loss function within the forward and backward substitution. The upper triangular matrix is scaled by the square root of the forgetting factor. Based on the obtained value of the loss function, we have updated weights using the weight updating blocks.

**Weight Updating:** Parameters of a wireless communication channel have to vary quickly, thereby weight updating should be performed at a higher rate than a statistic scenario. An alternative solution for updating the weight vector consists of directly computing the inverse of the correlation matrix using the MVDR algorithm. The primary goal of the MVDR beamforming technique is to minimize SINR expressed by the following formula [21,22],

(23) $$\mathrm{SIN}R≜\frac{{\left|\langle \left(C_{n}e^{jn.r_{n}}\right),(h\left(\phi_{n},\theta_{n}\right)\rangle \right|}^{2}}{{\left|\langle \left(C_{l}e^{jn.r_{n}}+v_{n}+i_{n}\right),(h\left(\phi_{n},\theta_{n}\right)\rangle \right|}^{2}}$$

The additive white Gaussian noise at outputs of the hybrid array antennas is denoted by v, such that the *n*-th entry of v, $v_{n}\approx CN(0$, $\sigma^{2}$). It is assumed that $\sigma^{2}=1$.

Hence, the minimum SINR is achieved by the MVDR beamforming technique when the following conditions are satisfied:

(24) $$\begin{array}{cc} \; & \min_{\alpha }\langle \left(C_{n}e^{jn.r_{n}}+v_{n}+i_{n}\right),(h\left(\phi_{n},\theta_{n}\right)\rangle \end{array}$$

(25) $$\begin{array}{cc} \; & \;s.t. \end{array}$$

(26) $$\begin{array}{cc} \; & \;\langle h^{H}\left(\phi_{n},\theta_{n}\right),C_{n}\left(\phi ,\theta \right)\rangle =1 \end{array}$$

where $h^{H}$ denotes the Hermitian matrix of the steering vector of h. Hence, the MVDR beamforming solution is expressed as

(27) $$w_{\left(MVDR\right)}=\alpha |h^{-1}{\left(\phi_{-n+l},\theta_{-n+l})\right|}^{2}C_{n}\left(\phi ,\theta \right)$$

The normalized constant of α in Equation (27), which does not have any effect on the SINR, can be omitted.

(28) $$\alpha =\frac{1}{{\left|\langle \left(C_{n}\right),\left(h^{-1}\left(\phi_{-n+l},\theta_{-n+l}\right)\right)\rangle \right|}^{2}}$$

**SVM Inner Product:** Hadamard multiplication (or element-wise multiplication) refers to the multiplication of array objects, which includes both of the Hadamard product of a∗b and the matrix product of a@b, as demonstrated in Figure 9 and Figure 11.

The tree sum with distributed pipelining takes a sequence of tasks in which each sequence includes its own steps and overlaps with other steps with different tasks in the execution time. The practical implementation of synchronizing the pipeline distributions can provide limitations, however performing a parallel merging on trees, as demonstrated in Figure 9, can significantly overcome the aforementioned constraints [17]. An increase in the number of pipeline stages results in a significant enhancement in the throughput of the FPGA and an increase in the operational clock frequency, however, an increase in the latency, which is not of practical interest. Therefore, we have to consider a trade-off between the performance enhancement in terms of the throughput of the FPGA and the operational clock frequency and latency by choosing an appropriate number of the pipeline stages. The proposed configuration of the pipelined inner product computations can drastically decrease delays in the mathematical operations, and power dissipation, and an area reduction.

## 6. Results of the QS-SVM-Based Beamformer on the FPGA Board

In this section, the proposed architecture for the setup and implementation includes the FPGA board, as the hardware unit, integrated with the MATLAB environment, as the processing unit or software unit. The proposed hybrid antenna array with bowtie elements [7] has been rigorously employed for the QS-SVM-based digital beamformer. In Figure 4, suppose that only one of three available incident signals in the environment is desirable. In order to separate and then classify the three signals, we should primarily retrieve directions of all the three incident signals using the DoA estimation, described in detail in [19,23,24,25,26]. Consequently, the proposed digital beamformer has delivered the synthesized beam pattern, as illustrated in Figure 11. The synthesized beam pattern consists of the two following tasks:

- Null steering for undesired signals by replacing nulls of the radiation pattern of the proposed hybrid antenna array in the detected directions of undesired signals. Hence, we can weaken significantly or eliminate undesired signals.
- Keeping the desired signal unchanged by exerting power with the 0dB level in the detected direction of the desired signal. We should neither strengthen nor weaken the desired signal, due to the following two reasons: (1) since the desired signals may include noise, jamming, interference, and other unwanted signals, any amplification in the desired signal results in magnifying noise and other unwanted signals, and (2) any reduction in the desired signal is not of practical interest.

## 7. Performance Evaluation of the FPGA-Based Beamformer

The discussion in the previous section focused on comprehensively demonstrating the deployment and setup of the digital beamformer. In the following section, we aim to evaluate and validate the digital beamformer performance. In this scenario, results-based monitoring allows for providing a framework to assess and evaluate the proposed digital beamformer performance in terms of (1) throughput evaluation, (2) average latency time, and (3) performance efficiency.

### 7.1. Throughput Evaluation

The Euclidean distance refers to the most well-known distance metric for deep learning as well as the classification process in machine learning. The Euclidean distance metric allows for measuring the distance between paired examples in the high-dimensional feature space. However, the utilization of Euclidean distance results in neither preserving the correlation of pairs nor enhancing the robustness of pairs. To overcome the aforementioned limitations, Tongtong Yuan and his research group in [27] have rigorously verified that the SNR distance is a more promising metric for measuring differences between pair features. Moreover, they extracted the SNR formulation for deep learning as the ratio of feature variance and noise variance. Since the throughput of the classification output is very much affected by SNR values, a drastic degradation in the SNR functionality causes a totally unacceptable throughput.

**Figure 12:**
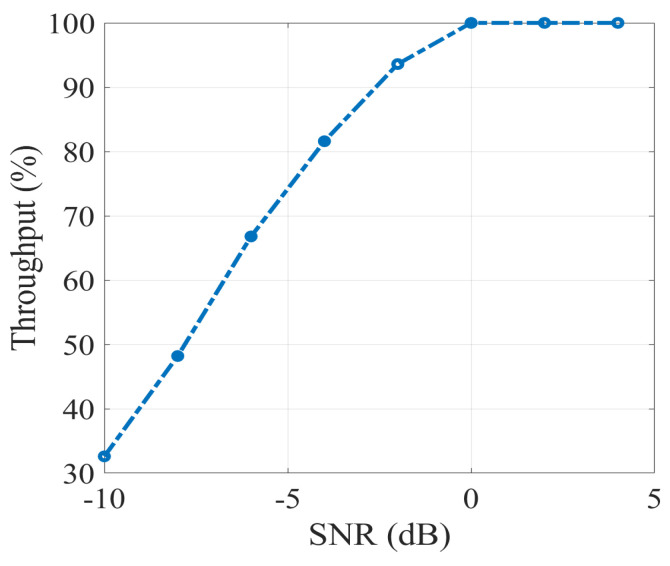
Variation of throughputs of the classification performance of the QS-SVM-based beamformer in terms of SNRs consistent with Figure 3.

Figure 12 illustrates that the throughput of the classification output has significantly increased in the presence of greater SNR values. Similar results of Figure 12 have been achieved in [27], in which the enlargement of inter-class distances for learned features resulted from the better performance of the SNR distance (or the higher throughput of the classification output) which is accompanied by an increase of SNR values.

### 7.2. Latency Evaluation

**Figure 13:**
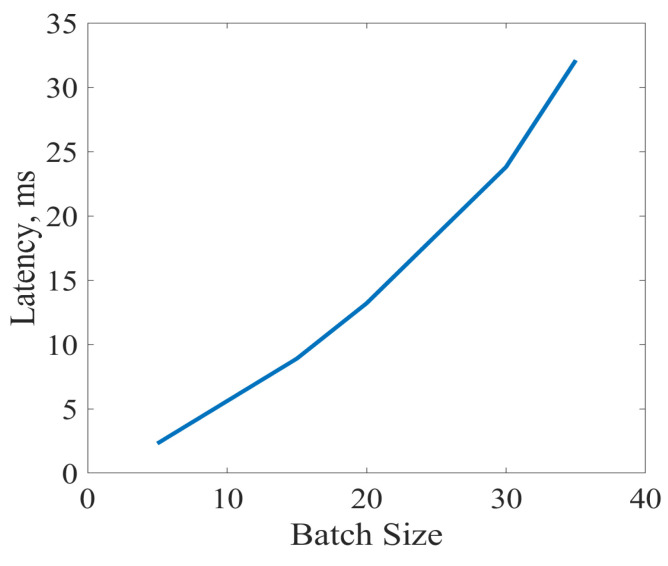
Latency analysis for different batch sizes consistent with Figure 3. Latency is presented in milliseconds and averaged over the test set of the network.

Latency allows for measuring the average time for processing a fixed-point dataset in different sizes of a batch. In the supervised ML, a batch size refers to the length of the training dataset in each iteration. In an ideal scenario, in which the FPGA integrated with a processor is working at speed, an average latency time is around or below 50 microseconds. Figure 13 has reported the simulation results on the average latency time of the implementation setup of Figure 3 in terms of batch sizes. Figure 13 ensures that the latency time is very much affected by the length of the fixed-point training data. In other words, an increase in the length of the training dataset is accompanied by a reduction in the processing time.

### 7.3. Performance Efficiency

Table 2 represents analogous results of the performance efficiency of the FPGA-based implementation with the hybrid antenna array with the two different types of array elements of Figure 1. In this scenario, the efficiency of the ML-based DoA estimator for the hybrid antenna array with bowtie elements outperforms its geometrical counterpart with dipole elements by more than 20%.

## 8. Conclusions

The kernel-free classifier of QS-SVM can effectively support non-linearly separable datasets without mapping them to any larger feature space in the DoA estimation. In addition, adding an extra regularizer to the objective function has promoted the capability of the QS-SVM technique for linearly-separable datasets of the DoA estimation. The MVDR beamforming technique is capable of updating weight vectors efficiently, but it has the weak performance innullsteering. We have demonstrated that the nullsteering performance has been strongly improved by performing an additional beamforming technique of LCMV, producing deep nulls with powers less than −10 dB in the directions of the two undesired signals.

The configuration of the antenna array, which has been employed for the proposed beamformer, can significantly affect the performance and functionality of the DoA estimation and beamforming techniques. The spatial distribution of the hybrid antenna array in the horizontal and vertical directions and the specific characteristics of its radiation pattern have significantly enhanced its capability in controlling the reception of desired signals. In the following, the software and hardware implementations of the QS-SVM-based beamformer have been fulfilled in the Mathworks’ Simulink visual programming environment and FPGA board, respectively. Details of the hardware implementation on the FPGA board have been fully demonstrated. The proposed optimization techniques incorporated by the hybrid array antenna arrays have resulted in a very strong beamformer with superior capabilities of nullsteering, a high throughput of about 100%, a low average latency, and a high-performance efficiency of more than 90%.

## Figures and Tables

**Table 1:** The design parameters for the proposed hybrid antenna array of Figure 1.

Parameters	Definition	Value
$N_{h}$	Number of elements of any circular loop	$N_{h}=20$
$Q_{h}$	Number of elements of any cylinder	$Q_{h}=40$
$M_{h}$	Total number of cylinders in the proposed array	$M_{h}=3$
$P_{h}$	Number of circular loops in the cylinder	$P_{h}=2$
$d_{v}$	Vertical spacing between two consecutive circular loops	$d_{v}=0.5\lambda$
$d_{r}$	Horizontal spacing between two consecutive circular loops	$d_{r}=0.5\lambda$
ϕ,θ	Maximum scanning angles	$\phi =45^{\circ },\theta =45^{\circ }$

**Table 2:** A comparison between the classification performance of the QS-SVM beamformer using the hybrid antenna array with bowtie elements, Figure 1, and its geometrical counterpart with dipole elements. The hybrid array antennas have been demonstrated in [7].

	Antenna array with bowtie elements	Antenna array with dipole elements
Performance efficiency of the proposed QS-SVM beamformer	96%	75%

## Abbreviations

The following abbreviations are used in this manuscript:

MDPI	Multidisciplinary Digital Publishing Institute
DOAJ	Directory of Open Access Journals
TLA	Three Letter Acronym
LD	Linear Dichroism
